# Modulation of Fibers to Motor Cortex during Thalamic DBS in Tourette Patients Correlates with Tic Reduction

**DOI:** 10.3390/brainsci10050302

**Published:** 2020-05-15

**Authors:** Pablo Andrade, Petra Heiden, Moritz Hoevels, Marc Schlamann, Juan C. Baldermann, Daniel Huys, Veerle Visser-Vandewalle

**Affiliations:** 1 Department of Stereotactic and Functional Neurosurgery, University Hospital of Cologne, 50397 Cologne, Germany; petra.heiden@uk-koeln.de (P.H.); mauritius.hoevels@uk-koeln.de (M.H.); veerle.visser-vandewalle@uk-koeln.de (V.V.-V.); 2 Department of Neurosurgery, University Hospital of Cologne, 50397 Cologne, Germany; 3 Department of Neuroradiology, University Hospital of Cologne, 50397 Cologne, Germany; marc.schlamann@uk-koeln.de; 4 Department of Psychiatry and Psychotherapy, University Hospital of Cologne, 50397 Cologne, Germany; juan.baldermann@uk-koeln.de (J.C.B.); daniel.huys@uk-koeln.de (D.H.); 5 Department of Neurology, University Hospital of Cologne, 50397 Cologne, Germany

**Keywords:** Tourette syndrome, deep brain stimulation, connectivity, tractography, imaging

## Abstract

Probabilistic tractography in Tourette syndrome (TS) patients have shown an alteration in the connectivity of the primary motor cortex and supplementary motor area with the striatum and thalamus, suggesting an abnormal connectivity of the cortico-striatum-thalamocortical-pathways in TS. Deep brain stimulation (DBS) of the centromedian nucleus–nucleus ventrooralis internus (CM-Voi complex) in the thalamus is an effective treatment for refractory TS patients. We investigated the connectivity of activated fibers from CM-Voi to the motor cortex and its correlation between these projections and their clinical outcome. Seven patients with TS underwent CM-Voi-DBS surgery and were clinically evaluated preoperatively and six months postoperatively. We performed diffusion tensor imaging to display the activated fibers projecting from the CM-Voi to the different motor cortex regions of interest. These analyses showed that the extent of tic reduction during DBS is associated with the degree of stimulation-dependent connectivity between CM-Voi and the motor cortex, and in particular, an increased density of projections to the presupplementary motor area (preSMA). Non-responder patients displayed the largest amount of active fibers projecting into cortical areas other than motor cortex compared to responder patients. These findings support the notion that an abnormal connectivity of thalamocortical pathways underlies TS, and that modulation of these circuits through DBS could restore the function and reduce symptoms.

## 1. Introduction

Tourette syndrome (TS) is defined as a chronic neuropsychiatric disorder characterized by sudden, uncontrolled, repetitive, stereotypical movements or vocalizations described as tics, typically starting during childhood [1]. The pathophysiology lying behind this disorder is still unclear. Some studies suggest a higher cortical excitability in the primary motor cortex [2], which could result from underlying alterations in the cortico-basal ganglia-thalamocortical (CBTC) loops [3]. TS patients have a higher functional disorganization and an increased global interaction in the CBTC circuits, suggesting a deficiency in brain [4]. Probabilistic tractography of TS patients have shown an alteration in the connectivity of the primary motor cortex and supplementary motor area with the striatum and the thalamus. However, these reports resulted in contradictory conclusions, with some studies showing a decreased connectivity [5,6], while others showed an enhanced connectivity [7,8].

More than half of the patients become tic-free or only present with mild symptoms after reaching early adulthood, therefore not needing further treatment [9]. In severe cases refractory to behavioral therapies and pharmacological treatment that persist during adulthood, deep brain stimulation (DBS) can be considered as a therapeutic option [10]. Up until now, there are several different targets along the CSPT circuit that have been explored in TS patients, the most commonly used being the thalamus, the globus pallidus internus (GPi), the globus pallidus externus (GPe), the anterior limb of the internal capsule/nucleus accumbens (ALIC/NA) and the subthalamic nucleus (STN) [11]. Nevertheless, among these targets, the majority of cases have been performed on the medial thalamus and the GPi. Within the medial thalamus, various possible targets have been described, the most frequently used being the centromedian nucleus–nucleus ventrooralis internus (CM-Voi) and the centromedian nucleus–parafascicular (CM-Pf) complexes [12]. The clinical outcome of TS DBS in these targets is relatively variable within the same cohorts, with none showing a clear advantage over the others [13,14].

In recent years, diffusion tensor imaging (DTI) has emerged as a useful tool for optimizing targets in DBS for different anatomical targets. DTI allows to display fibers projecting from the tissue activated at a nominated target to different cortical and subcortical regions through tractography [15]. Previous studies on different neurological and psychiatric disorders have shown direct correlations between connectivity patterns and clinical outcome in patients, for instance, in obsessive-compulsive disorder (OCD) [16], Parkinson’s disease [17] and tremor [18]. The aim of this study is firstly to examine the connectivity of activated fibers in TS patients during CM-Voi DBS and secondly, to elucidate if connectivity patterns are correlated with a better clinical outcome after long-term CM-Voi DBS.

## 2. Materials and Methods

### 2.1. Patients and Study Design 

We retrospectively analyzed seven patients who underwent surgery for bilateral thalamic DBS for TS at the University Hospital of Cologne between 2016 and 2018. Patients were diagnosed based on the diagnostic and statistical manual of mental disorders fifth edition and the Tourette syndrome diagnostic confidence index [1]. The clinical evaluation was assessed using the Yale Global Tic Severity Scale (YGTSS), which was performed before, and six months after the surgery. This study was approved by the Medical Ethical Committee of the University Hospital of Cologne (Code 19-1421). All subjects granted their consent according to the Declaration of Helsinki. 

### 2.2. Stereotactic Surgery 

The CM-Voi complex was targeted for stimulation with standard coordinates (midpoint AC/PC line coordinates: x= +/− 5 mm, y= −4 mm, z= 0 mm), adapted to the patient’s anatomy and with trajectory along the longest axis of Voi, based on preoperative magnetic resonance imaging (MRI) fused with stereotactic computed tomography (CT) scan with the atlas of the human brain (Schaltenbrand Atlas) overlaid (Cranial Software, version StealthStation™ S7, Medtronic, Minneapolis, MN, USA). Surgery was performed under analgosedation with intraoperative microelectrode recordings. All patients received quadripolar electrodes (Medtronic 3389) bilaterally, stereotactically guided. The accurate localization of the electrodes was confirmed via postsurgical CT scan. For optimal stimulation, the frequency, voltage and pulse width were individually adapted for best results. 

### 2.3. Fiber Tracking

Based on previous studies [5,8], we selected the primary motor cortex (M1), the supplementary motor area (SMA) and pre-SMA as our seed regions. We defined M1 manually in the ICBM 152 MNI 2009b asymmetrical cortical atlas and used the Human Motor Area Template [19] for SMA and preSMA. These cortical mask regions were transferred to each patient’s T2 sequence using FLIRT from the FSL (FMRIB Software Library, www.fmrib.ox.ac.uk/fsl) v6.0 program for affine linear matching, followed by deformable registration by the advanced normalization tools (ANTs, http://stnava.github.io/ANTs/) [20]. The seed regions were subsequently manually adjusted using the ITK-SNAP desktop app to each patient’s individual anatomy based on T1 sequences on the preoperative MRI [21].

Using the open-source software Lead-DBS (www.lead-dbs.org, Charité University Hospital, Berlin, Germany), we coregistered and normalized the preoperative MRI scans and the postsurgical CT scan to MNI space [22]. Afterwards, we reconstructed the electrodes and the active contacts. Using the model described by Dembek and colleagues [23], we estimated the area stimulated by the active contacts (VTA: volume of tissue activated) based on the stimulation parameters, with a general heuristic electric-field threshold of 0.2 V/mm. We transferred the VTAs, using the same transformation procedure as described above, into patient space. The DTIs were corrected for distortions and subject movement using FUGUE from FSL tools [24,25,26,27]. After this, we performed the probabilistic fiber tracking from the VTAs to the ipsilateral hemisphere as well as the ipsilateral M1, SMA and preSMA using the Oxford Centre for Functional MRI of the Brain (FMRIB), FSL probtrackx2 program [28,29].

For further analysis, we applied the seed regions as binary masks on the respective tracks using the fslmaths tool and quantified the mean and maximum intensity of voxels localized within the seed regions. 

### 2.4. Statistical Analysis

All data were analyzed using GraphPad Prism software (version 5, GraphPad Software, San Diego, CA, USA) with *p*-values under 0.05 considered as significant. In order to estimate the correlation between clinical improvement in the YGTSS and the fiber tract values, data were analyzed using a linear regression analysis. A Wilcoxon signed-rank test was performed to compare pre- and post-operative clinical YGTSS score differences between the samples. A Spearman rank’s test was performed to establish correlation analyses between different parameters. A power calculation was performed in order to calculate the amount of TS patients required for a significant statistical analysis (90% power, 0.05 alpha).

The data that support the findings of this study, such as the DBS MRI datasets, are not publicly available due to data privacy regulations, but are available from the corresponding author upon reasonable request.

## 3. Results

### 3.1. Demographic and Clinical Characteristics

Demographic and individual clinical data from patients included in this study (*n* = 7, six men and one woman, age mean 30 years SD = 9.1) are presented in Table 1. Disease onset ranged from three to ten years of age. Comorbidities were present among the participants with the most frequent conditions including OCD and depression (*n* = 2, 28% each). Disability and impairment were severe in every patient with a mean total preoperative YGTSS score of 90 (SD = 8.7). The mean YGTSS score six months postoperatively was 52 (SD = 21.4), with a significant mean improvement in YGTSS score of 43.5% (SD = 18.9, *p* < 0.001) pre- versus post-operatively. Clinical response being defined as an improvement of at least 35% on the YGTSS, five patients were considered responders (mean improvement in YGTSS 52.7% with a range of 43.7–71.8%) and two as non-responders (mean improvement in YGTSS 20.5% with a range of 11–30%).

### 3.2. Neuroimaging Analysis

#### 3.2.1. Generation of Fiber Tracts and Cortical Projections

**Figure 1:**
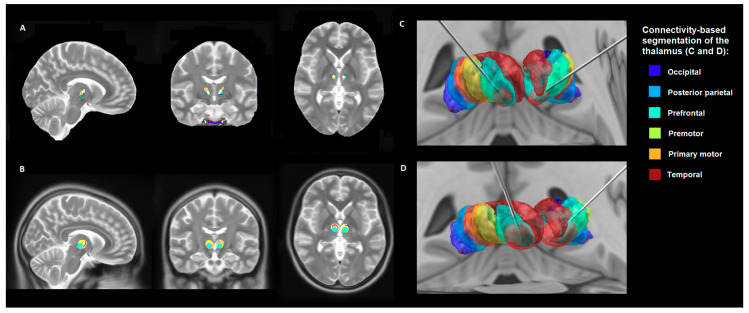
Anatomical location of electrodes and volume of tissue activated (VTAs) within the thalamus. Overview of bilateral electrodes in the centromedian nucleus–nucleus ventrooralis internus (CM-Voi) complex (**A**) and cluster for bilateral VTAs of all patients on the MNI space (**B**) in sagittal, coronal and axial views. Volume of tissue activated three-dimensional representation shown in light red for the best clinical responder in the cohort, only patient 1 represented (**C**). Volume of tissue activated three-dimensional representation shown in light red for the patient with the worst clinical outcome in the cohort, only patient 7 represented (**D**). Thalamic segmentation according to cortical connectivity is shown in different colors in C and D in order to exemplify the position of the implanted electrodes [30].

**Figure 2:**
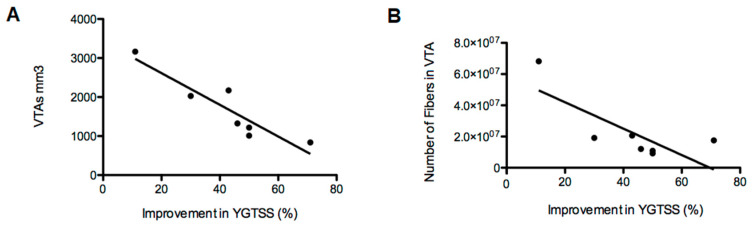
Scatter plot depicting the correlation between postoperative improvement of the Yale Global Tic Severity Scale (YGTSS) after six months expressed in percentage and the volumetric measurements of the generated VTAs ((**A**), *p* < 0.01, *R* = −0.84) and the numbers of fibers in the VTA that project to the cortex ((**B**), *p* < 0.05, *R* = −0.58). Data shown in this figure represent individual patient analysis (*n* = 7). For a segmented analysis of each hemisphere (*n* = 14), please consult Figure A1.

The individual location of bilateral electrodes and the generated VTAs of all patients are shown in Figure 1A,B, respectively. A clear difference is seen among the generated VTAs between responders and non-responders, as shown in Figure 1C,D, respectively. Non-responders presented with significant (*p* < 0.05) higher VTA-volumes (mean volume 2597 mm^3^, SD = 804) compared to responders (mean volume 1313 mm^3^, SD = 515). Furthermore, a direct comparison between volumetric values of individual VTAs and the percentage of improvement in the YGTSS six months after surgery showed a significant negative correlation in all participants (*p* < 0.01, *R*= −0.84), as shown in Figure 2A. Thus, the patients with better clinical outcomes showed smaller magnitudes in the generated VTAs. Derived from these results, we analyzed the amount of active fibers involved in the corresponding VTAs that projected to all cortical areas. This analysis showed a significant negative correlation between the amount of cortical fiber tracts and the percentage of improvement in the YGTSS six months after surgery (*p* < 0.05, *R* = −0.58), as displayed in Figure 2B. Furthermore, the non-responder patients presented with larger amount of fibers projecting to the cortex with higher variability between individuals (mean number of fibers 45,452,500, SD = 35,585,148) when compared to the responder participants (mean number of fibers 15,038,000, SD = 4,976,307). Therefore, the patients with better clinical outcomes displayed a general reduced cortical fiber activation in the whole brain when compared to the non-responders.

#### 3.2.2. Parcellation of Seed Regions

**Figure 3:**
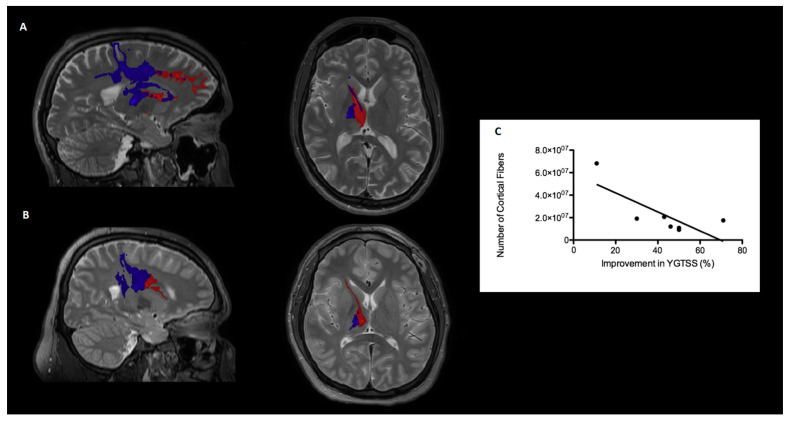
Comparison of structural connectivity between a non-responder (**A**) and a good responder (**B**) patient, in sagittal and axial views. All fibers traversing the VTAs that connect into the preSMA (presupplementary motor area), SMA and M1 (primary motor cortex) cortices are shown in blue, while the rest of the fibers that project to other parts of the cortex are shown in red. Correlation between the clinical response and the total amount of cortical projections into nonmotor seed regions ((**C**), *p* < 0.05, *R* = −0.60).

Parcellation of the seed regions of interest in the motor cortex (preSMA, SMA and M1) showed that non-responder patients displayed the largest amount of active fibers, but projecting to cortical areas different from the seed regions than responder patients. This means that, although cortical connectivity in non-responders was generally increased, it was primarily projecting to nonmotor areas, as shown in Figure 3. These active fibers projected in its majority to different areas of the prefrontal cortex, as shown in Figure 3A. Responders displayed generally less modulated connectivity compared with non-responders, which was highly clustered towards the preSMA, SMA and M1 cortices, as shown in Figure 3B. Moreover, the total amount of cortical fibers originated in the VTAs that projected to nonmotor seed regions showed a negative correlation with the percentage of improvement in the YGTSS six months after surgery (*p* < 0.05, *R* = −0.60), as displayed in Figure 3C.

#### 3.2.3. Density of Fibers in the Motor Cortex

**Figure 4:**
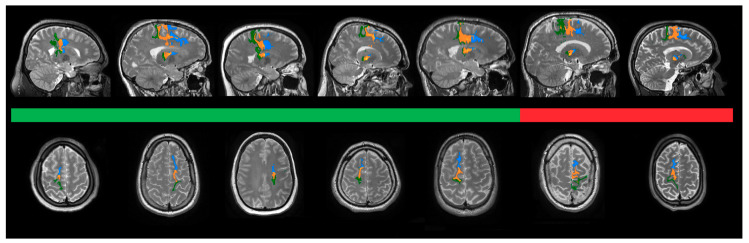
Connectivity-based segmentation of the seed areas. Representative tractographies of each patient. Images are shown in patient-space, where the most significant density of fibers was observed. Therefore, the most symbolic MRI cut of each patient was depicted, including the most representative hemisphere in each individual. Fibers originated from the VTAs that connect to M1 are shown in green, whereas fibers connecting to SMA and preSMA are shown in orange and blue, respectively. Patients are separated in responders (green bar) and non-responders (red bar) in descending order from the best to the worst clinical outcome from left to right. Independently from their clinical outcome, all patients showed thalamic connectivity to the seed areas.

**Figure 5:**
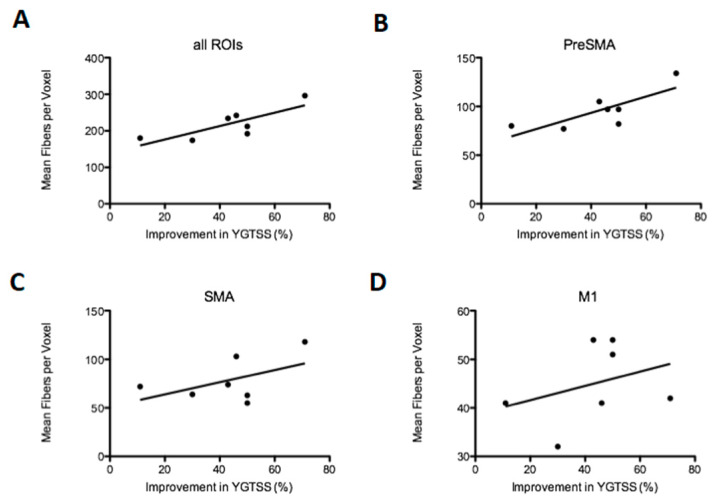
Correlation between postoperative improvement of YGTSS after six months expressed in percentage and the mean of fibers per voxel located within the cortical seed regions. All regions of interest (ROIs, *p* < 0.05, *R* = 0.63) summed together including the preSMA, SMA and M1 are shown in (**A**), while the independent seed regions are shown separately in (**B**) (preSMA, *p* < 0.05, *R* = 0.61), (**C**) (SMA, *p* > 0.05, *R* = 0.25) and (**D**) (M1, *p* >0.05, *R* = 0.11). Data shown in this figure represent individual patient analysis (*n* = 7). For a segmented analysis of each hemisphere (*n* = 14), please consult Figure A2.

The tractography of the three seed regions analyzed independently showed that all patients displayed active fibers projecting to all cortices including preSMA, SMA and M1, as shown in Figure 4. The density of the projections to the motor cortex was analyzed by investigating the mean amount of activated fibers per voxel inside the seed regions. This analysis showed a significant positive correlation between the mean density in all seed regions together (*p* < 0.05, *R* = 0.63) as well as in preSMA (*p* < 0.05, *R* = 0.61) in association with the percentage of improvement in the YGTSS six months after surgery, demonstrating a higher density of projecting fibers within these areas in patients with better clinical outcomes, as shown in Figure 5A,B. SMA and M1 showed both a positive tendency on the mean density, however, it was not significant when analyzed independently (*p* > 0.05 both, *R* = 0.25 and *R* = 0.46, respectively).

## 4. Discussion

In the present study we investigated the connectivity between the CM-Voi complex in the thalamus and the motor cortex during DBS in TS patients. Our findings showed that an increase in the density of fiber projections to the seed regions of the motor cortex, including preSMA, SMA and M1, was accompanied with better clinical outcomes. These results support the concept that abnormal structural connectivity of thalamo-cortical pathways in TS may be involved in the generation of motor and vocal tics [8]. More specifically, activation of fibers projecting to the preSMA was significantly higher in responder patients. In line with these findings, previous studies have suggested that tic severity is correlated with activations in the premotor cortex (SMA and preSMA) [31]. Although the function of these cortical areas in TS is still to be determined, it has been proposed that its role in planning of movements, motor inhibition and therefore voluntary tic suppression may be related to symptom reduction [32,33]. Furthermore, there is evidence that the urge to tic is associated with the SMA region, thus DBS for TS may also act by reducing pathological urges to tic [34]. Although previous studies have reported apparent conflicting results about connectivity in TS patients, it is noteworthy to mention that certain differences in the design between studies can be observed [5,6,7,8]. For instance, Cheng and colleagues reflect if progression of the disease can play a role in reducing connectivity patterns in TS, since they studied a cohort of adult patients with a mean disease duration of almost three decades [5]. Nevertheless, Makki and colleagues reported comparable results with lower probability of connection between caudate nucleus and anterior-dorsolateral-frontal cortex on the left hemisphere in children with TS [6]. Another crucial difference between these two reports was the presence of comorbidities. While Cheng et al. selected a cohort of TS patients without psychiatric comorbidities, Makki et al. studied a group of individuals with accompanying psychiatric behavior like OCD. The presence of other disorders may complicate the analysis of these results, since multiple circuits may involve several cortical structures simultaneously. Although our results confirm the relevance of these cortical structures, there are still certain differences to these studies. Contrarily, Thomalla and colleagues showed enhanced connectivity between the thalamus and the SMA [7]. In this study, the cohort may be more comparable to our patients, where younger adult patients with similar duration disease duration where included. However, our group of TS patients was fairly heterogeneous, with an almost equally divided sample of patients with comorbidities and without them. Consequently, medication between participants was also quite diverse in our group. On the contrary, Thomalla et al. included TS patients with no psychiatric comorbidities and no medications at the inclusion to the study. Nevertheless, the same authors of this report discussed the very selected group of patients with these unique characteristics, which may not necessarily represent the majority of the TS population. In this regard, when we analyze the patients included in our study, two cases were also diagnosed with OCD, one of the most common comorbid conditions in TS. This finding could be interpreted by itself as a better representation of comorbidities of this disorder in our study than the ones reported in other studies. It could also be interpreted as a confounding factor, since these two patients with OCD had the worst clinical outcome. The connectivity profile of these two cases interestingly followed a similar pattern, showing a clear difference with the patterns of responders. Recapitulating, all these alterations in connectivity probably illustrate a process of adaptation between pathological input at diverse stages of the disease and the capacity of compensation according to the individual variables of each patient. In this context, it is relevant to mention that the connectivity displayed in our study is a stimulation-dependent connectivity, while the above-mentioned studies are based on structural connectivity paradigms. For this reason, it was of crucial importance that our DTIs were performed under anesthesia. This way we avoided movement-associated artifacts, since the slightest movement in TS patients could generate alterations in tractographies.

We also demonstrated that non-responders displayed larger amount of active fibers than responder patients, but projecting to cortical areas different from the seed regions. These findings suggest that diffuse stimulation of several cortical areas simultaneously may have therapeutic implications on these patients. Since non-responder patients also displayed projections into the preSMA, SMA and M1 cortices, (however, with a lower density than the good responders) these findings may suggest that a more selective connectivity to these seed regions can reproduce better clinical outcomes. These observations translated directly into the size of the generated VTAs and the amount of fibers activated by these VTAs that projected into the entire cortex (preSMA, SMA, M1 and others). This assessment showed a clear negative correlation between higher VTAs volumes and the improvement in the YGTSS scores. Therefore, the consequence of these larger VTAs was the activation of a larger quantity of fibers that projected to the general cortex. Previous studies in different psychiatric disorders have shown a similar negative effect by an extensive activation of nonrelevant fibers during stimulation of the anterior limb of the internal capsule and nucleus accumbens in OCD patients [16]. In this study, the authors suggest that the activation of nonrelated structures may counteract the positive effect reached by the targeted fibers. In order to validate this concept, after we finished the interpretation of our results, we reanalyzed our data using fixed-size VTAs of 1, 2 and 3 Volts in all patients instead of the actual individual parameters of each subject. This analysis showed the same connectivity patterns of increased fiber projections to the general cortex and not primarily to the preSMA, SMA and M1 cortices in non-responders, therefore, demonstrating that the amount of activated fibers would ultimately be the most relevant factor, and not exactly the size of the VTAs. Although larger VTAs could activate more fibers, apparently this principle is dependent on the exact anatomical area that is stimulated. Moreover, not only the amount of fibers projecting to relevant areas may be critical for a positive outcome, but also the capacity to exclusively modulate the areas of interest. In order to achieve this objective, a more precise anatomical location of the active contacts within the thalamus may prove to be an essential factor. Our target is based on the positive clinical effect on Tourette symptoms of thalamotomies performed by Hassler and Dieckmann in 1970. Hassler and Dieckmann performed more than ten lesions per hemisphere, mainly in the median and intralaminar thalamic nuclei as well as the inner part of the ventral oral thalamic nucleus [35]. The rationale for the first DBS case was to strategically define one target so that the different nuclei lesioned by Hassler could be modulated by implantation of one lead per hemisphere [36]. This target was chosen at the anterior part of the centromedian nucleus (as part of the intralaminar thalamic nuclei), at its medial border with the medial thalamic nuclei, and along the Voi. DBS of this specific target has proven to be effective in a randomized controlled trial [37].

Comparable clinical results have been reported in up to four different targets within the CM thalamic region [12], however anatomical differences between these nuclei should be taken into consideration. A recent study with a similar setting, in this case targeting the CM-Pf (parafascicular) region, showed a high variability between the clinical outcome and the connectivity [38]. In this report, the cortical projections associated with better clinical responses were the right frontal middle gyrus, the left frontal superior sulci region and the left cingulate sulci region. None of these regions were particularly involved in our study, suggesting a potential difference between the connectivity of the CM-Voi and the CM-Pf complexes.

A major limitation of our study is the limited size of our sample, in the context of such a heterogeneous population. As we mentioned before, although this factor may represent more properly the general TS population, in our case it could also fragment the data and extrapolate the outlier data. Therefore, larger cohorts with similar conditions and long-term follow-ups even after initial clinical failure are required to validate the findings of our study. However, to our knowledge, this is the first time that TS patients with ongoing stimulation of the CM-Voi have been investigated with stimulation-dependent tractographies based on high-definition dMRI data.

## 5. Conclusions

To summarize, we demonstrate that the extent of tic reduction in TS patients after CM-Voi DBS is associated with the degree of connectivity between the stimulated area and the motor cortex. In particular, an increased density of projections to the preSMA, SMA and M1 areas correlates with better clinical outcomes after chronic DBS. These findings reinforce the theory that an abnormal structural connectivity of thalamo-cortical pathways underlies TS, and therefore the modulation of these circuits through DBS could reduce motor and vocal tics.

## Figures and Tables

**Table 1:** Demographic Table. Summarizes the clinical outcome of each patient six months after surgery, individual deep brain stimulation (DBS) parameters including the active contacts responsible for stimulation, comorbidities and medication. YGTSS—Yale Global Tic Severity Scale, OCD—obsessive compulsive disorder, THC—Tetrahydrocannabinol.

**Pat. Number**	**Gender**	**Age**	**YGTSS Preop**	**YGTSS Postop**	**Improvement (%)**	**Active Contacts**
Pat. 1	Male	22	78	22	71.8	0 and 1
Pat. 2	Male	32	89	44	50.6	2 and 3
Pat. 3	Female	26	95	47	50.5	1 and 2
Pat. 4	Male	37	81	43	46.9	2 and 3
Pat. 5	Male	20	87	49	43.7	2 and 3
Pat. 6	Male	46	100	70	30.0	2 and 3
Pat. 7	Male	27	100	89	11.0	1 and 2
**Pat. Number**	**Freq. Hz**	**Pulse Width µs**	**Ampl. V**	**Comorbidity**	**Medication**	
Pat. 1	130	90	3.2	None	Aripiprazole 10 mg, Citalopram 30 mg	
Pat. 2	130	90	3.3	None	Tiapride 800 mg, Quetiapine 150 mg	
Pat. 3	100	90	3.0	Depression	Escitalopram 10 mg, THC 5 mg, Risperidone 4 mg, Quetiapine 25 mg	
Pat. 4	110	90	4.1	Opiate dependence (in substitution)	Methadone 28 mL	
Pat. 5	100	150	5.2	None	None	
Pat. 6	80	150	5.0	Depression, OCD	Aripiprazole 5 mg, Pipamperone 40 mg, Quetiapine 200 mg	
Pat. 7	80	300	4.7	Impulse control disorder, OCD, anxiety disorder	Clonazepam 1 mg, Pregabaline 300 mg, Risperidone 2 mg, THC 15 mg, Venlafaxine 225 mg	

**Figure A1:**
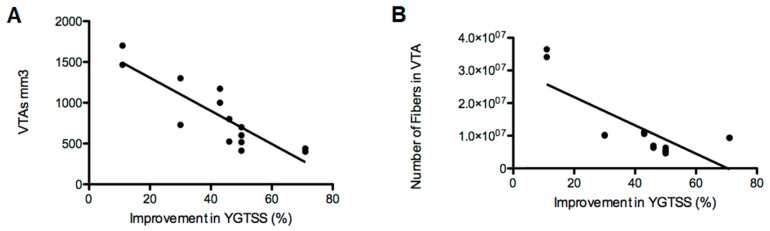
Scatter plot depicting the correlation between postoperative improvement of YGTSS after six months expressed in percentage and the volumetric measurements of the generated VTAs ((**A**), *p* < 0.001, *R* = −0.74) and the numbers of fibers in the VTA that project to the cortex ((**B**), *p* < 0.01, *R* = −0.58). Data shown in this figure represent a segmented analysis of each hemisphere (*n* = 14).

**Figure A2:**
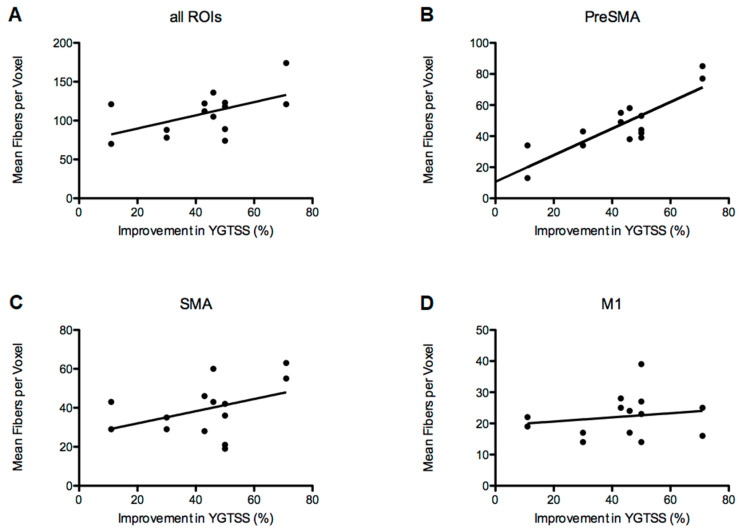
Correlation between postoperative improvement of YGTSS after six months expressed in percentage and the mean of fibers per voxel located within the cortical seed regions. All regions of interest (ROIs, *p* < 0.05, *R* = 0.32) summed together including the preSMA, SMA and M1 are shown in (**A**), while the independent seed regions are shown separately in figures (**B**) (preSMA, *p* < 0.001, *R* = 0.71), (**C**) (SMA, *p* > 0.05, *R* = 0.16) and (**D**) (M1, *p* > 0.05, *R* = 0.07). Data shown in this figure represent a segmented analysis of each hemisphere (*n* = 14).
